# Moving to and Dying in a Nursing Home Depends Not Only on Health – An Analysis of Socio-Demographic Determinants of Place of Death in Switzerland

**DOI:** 10.1371/journal.pone.0113236

**Published:** 2014-11-19

**Authors:** Damian Hedinger, Julia Braun, Ueli Zellweger, Vladimir Kaplan, Matthias Bopp, for the Swiss National Cohort Study Group

**Affiliations:** 1 Epidemiology, Biostatistics and Prevention Institute, University of Zurich, Zurich, Switzerland; 2 District Hospital Freiamt Muri, Muri, Switzerland; University Hospital Lausanne, Switzerland

## Abstract

**Background:**

In developed countries generally about 7 out of 10 deaths occur in institutions such as acute care hospitals or nursing homes. However, less is known about the influence of non-medical determinants of place of death. This study examines the influence of socio-demographic and regional factors on place of death in Switzerland.

**Data and Methods:**

We linked individual data from hospitals and nursing homes with census and mortality records of the Swiss general population. We differentiated between those who died in a hospital after a length of stay ≤2 days or ≥3 days, those who died in nursing homes, and those who died at home. In gender-specific multinomial logistic regression models we analysed N = 85,129 individuals, born before 1942 (i.e., ≥65 years old) and deceased in 2007 or 2008.

**Results:**

Almost 70% of all men and 80% of all women died in a hospital or nursing home. Regional density of nursing home beds, being single, divorced or widowed, or living in a single-person household were predictive of death in an institution, especially among women. Conversely, homeownership, high educational level and having children were associated with dying at home.

**Conclusion:**

Place of death substantially depends on socio-demographic determinants such as household characteristics and living conditions as well as on regional factors. Individuals with a lower socio-economic position, living alone or having no children are more prone to die in a nursing home. Health policy should empower these vulnerable groups to choose their place of death in accordance to needs and wishes.

## Background

The place of death is often considered as an important indicator of life quality in end-of-life care. [1], [2] In the early 20th century, most people died at home, but since the mid-20th century, the majority of people in industrialized countries die in health care institutions. [3] In spite of overwhelming individual preference for dying at home [4], [5] about two thirds of all deaths occur in acute care hospitals or long-term care facilities. [2], [3], This “hospitalization of death” [7] or “medicalization of dying” [1] is often considered as undesirable. [8] The divergence between preferences and reality makes place of death and the organization of end-of life care an important issue in medical and social research. Nevertheless, some researchers dispute the advantage of death at home compared to death in an institution. Dying at home can be very stressful in some situations for those involved, [9] especially when people need a lot of care. However, most authors agree that a “good death” should be a death that is - regardless of the place - in accordance with the patient’s and his or her family’s wishes. [3], [7], [10] Therefore, it is important to gain a better understanding of the reasons why people die where they do.

There is evidence that health care expenditure is highest at the end-of-life, and even increasing during the last months of life. [11], [12] Estimates show that 27% of Medicare’s annual budget is spent for the care of patients in their last year of life. [13] However, this “proximity to death” hypothesis is challenged by many confounders such as age, functional impairment, accumulation of chronic medical conditions and socio-economic characteristics of the deceased individuals. [14], [15] Indeed, place of death has a considerable impact on health care costs, with end-of-life care being more expensive in hospitals than in nursing homes or at private settings [16].

In some European countries, different models of palliative care have been developed. Their effectiveness and their impact on care at the end-of-life varies substantially. [17] It seems that home-based palliative care models increase the quality of end-of-life care. [18] Unfortunately, the situation of palliative care in Switzerland is difficult to assess because of regional variation and lack of representative data. [19] Specific hospices with in-patient facilities are scarce in Switzerland and cannot be reliably identified from the available data sources. Regional differences related to place of death have been described in few studies. The proportion of deaths occuring in hospitals in 1979/80 varied widely between 27% and 81%, [20] and that of deaths occurring at home 2007–11 between 22% and 33%. [16] It was argued that the proportion of deaths in hospital beds decreased when the access to primary care providers and nursing home beds increased. [16], [20] However, without information about socio-demographic characteristics a proper adjustment was not possible. Previous studies from other countries suggest that individual characteristics including old age, poor health status, living alone, female gender and low socio-economic status increase the risk for dying in an institution [21]–[23].

In our study, we extracted a variety of socio-demographic characteristics from the Swiss National Cohort, and explored their association with place of death adjusting for health related factors. In addition to socio-economic position (SEP) and age, we included other socio-demographic and several family and housing variables. [22], [23] Given the substantial regional variation in health care use and health-related outcomes in Switzerland, [20], [24] we also adjusted for regional characteristics. We hypothesized that people with a low SEP or poor living and household conditions have a higher probability to die in a nursing home.

## Data and Methods

### Data

We extracted data from three different sources covering all individuals living in Switzerland:

1. The Swiss National Cohort (SNC, www.swissnationalcohort.ch) is an anonymous linkage of census, mortality and emigration records. [25] Decennial censuses were conducted between 1850 and 2000 and registered sex, age, date of birth, nationality, marital status, religious affiliation, educational level and living arrangements. In addition, a variety of information about housing was registered. The 1990 census included for the first time the exact date of birth, which allowed linking census and mortality data.
2. The medical statistics of the Swiss hospitals (MedStat), administered by the Swiss Federal Statistical Office, register all hospital discharges since 1998. [26] Data collection is mandatory for all Swiss hospitals. Since 2002, the data source is more or less complete for the entire country. Besides medical information like diagnoses and treatments, [27] age, sex, place of residence, admission and discharge date and administration information like medical-insurance specifications are available.
3. The statistics of socio-medical institutions (SOMED) encompass nursing homes, homes for disabled, institutions for addiction patients and institutions for people with social problems. It is administered by the Swiss Federal Statistical Office and mandatory for all socio-medical institutions. [28] The institutions report limited patient information such as year of birth, sex, ZIP-code of residence, date of admission and discharge.

**Figure 1:**
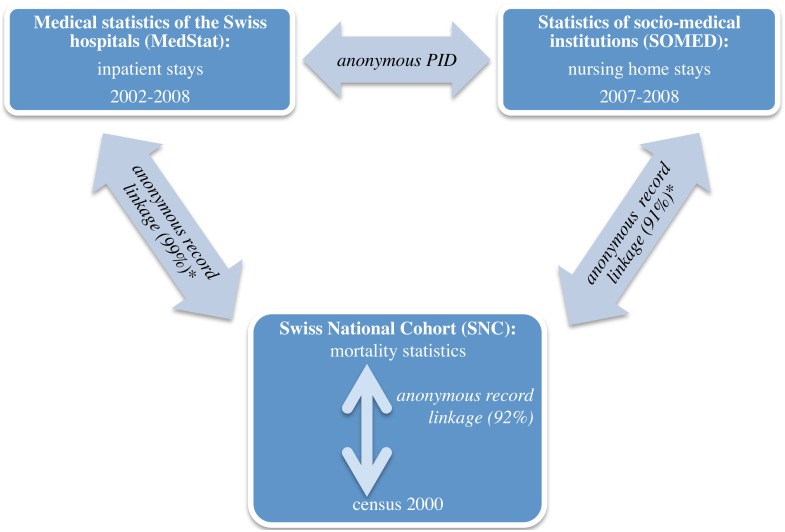
Three data sources and linkage. *using exact date of birth and death, sex, place of residence. Linkage success in brackets.

All three data sources are fully anonymized. For the purpose of privacy protection there is no common personal identification number that would allow to directly link the three data sources on an individual level. A common person identifier is only available for two of the three data sources (MedStat and SOMED). The linkage with the third source (SNC) had to rely on common identification variables such as place of residence, date of birth and date of death (see also Figure 1). In MedStat, the full date of death and date of birth are only available for those who died during their hospital stay. In SOMED, the full date of death and the year of birth are available for those who deceased. [29], [30] The linkage to the SNC was therefore only possible for those who died in an institution. [25] Figure 1 shows the linkage process of the three data sources.

The first step was MedStat data cleaning (e.g. correct bad encoding, duplicate records) from 2002 to 2008 of persons born before the 2000 census (N hospitalizations before: 12,629,525, N after: 12,234,964). Linkage of MedStat and SNC started with hospital deaths between 2002 and 2008 (N = 165,843 deceased patients). Of these, N = 163,971 (98.9%) could be successfully linked to a record in the mortality statistics. 95.1% of the links were totally concordant and 3.8% partially concordant (e.g., a record in MedStat and SNC with identical sex, birth date, and region of residence [around 600 regions in Switzerland] but some minor discordance regarding date of death, was assumed to belong to the same person). The data quality of MedStat was comparable for the time period from 2002 to 2008. The linkage of mortality statistics with SOMED started with data cleaning of the inpatient stays from 2006 to 2008. Because there were records from previous years and missing codes (mainly for 2006 cases), the number of retained cases dropped by more than 50%: From N = 486,681 to N = 229,367 cases. For a lot of the 2006 cases, which had also a 2007 record from the same institution, the anonymous person number from 2007 could be used. The linkage rate with the mortality records for the years 2007 and 2008 was about 91% [30] (N total: 44,124, N with successful link: 40,168). 65.1% of SOMED cases could be linked with totally concordant criteria and additional 25.9% with partially discordant criteria. Finally, 92% of the mortality records could be linked to a 2000 census record.

We restricted the study population to elderly people born before 1942 (≥65 years) and who had died in 2007 and 2008. We excluded people who already lived in a collective household (mostly nursing homes) during the 2000 census. Thereafter, we cleaned the data again (e.g. clean-up of duplicate records), leading to a final study population of N = 85,129 people (N = 39,798 men, N = 45,331 women).

### Study design

We categorized the outcome variable place of death as nursing home, hospital long (length of stay ≥3 days), hospital short (length of stay ≤2 days) and private home. The latter encompassed all deaths that could not be linked to a hospital or a nursing home record. We stratified our analysis by gender because there are fundamental differences between men and women regarding the place of death [2], [16], [21], [23], [31].

The independent variables were grouped into individual, familial/housing and structural/regional attributes. As control variables we included age and nationality (Swiss or foreign) as well as important causes of death: malignant neoplasms (ICD 10: C00-C99), coronary heart disease (I20-I25), chronic obstructive pulmonary disease (COPD, J40-J47), dementia (F01, F03, G30), stroke (I60-I69) and all other causes. Marital status was assessed at the time of death (never married, married, widowed and divorced). From the 2000 census we derived the educational level according to the International Standard Classification of Education (ISCED), version 1997: no secondary education completed (ISCED 0-1), lower secondary (“low”, ISCED 2), post-secondary non-tertiary (“medium”, ISCED 3-4), and tertiary education (“high”, ISCED 5). Also from the 2000 census we extracted information on homeownership (owner-occupier household yes vs. no), number of children (independently assessed for men and women), living arrangement (single-person- vs. multi-person- household), number of rooms per person in the flat or house. Place of residence was categorized into the three main Swiss language areas, namely German, French and Italian. In order to account for geographical variation in nursing home bed availability, we included a variable with the density of nursing home beds per 100 inhabitants aged 65 years or older in 2010 for small and homogenous regions (106 regions for the whole country).

### Statistical methods

For descriptive analysis, we calculated means, frequencies and proportions of the respective variables. Separate multinomial logistic regression models for both men and women, yielding estimated relative risk ratios (RRR), [32] were used to assess the impact of several independent variables on the relative risks for dying at different places of death. For better understanding of the interpretation of RRRs, we give an easy example using a hypothetical estimated RRR of 1.5 for a person in category B of some categorical variable: This would mean that in comparison to somebody in category A (reference category) of that variable, the relative risk of dying in an institution compared to dying at home is 1.5 times higher. As the interpretation of the results is not always easy, especially for categorical variables, we additionally provide graphical representations of the predicted probabilities of dying at different locations for a selection of the independent variables.

The variables age, nationality and language region were included in the model, because we felt them to be definite confounders. Age was modelled as restricted cubic spline with five knots (at 69, 78, 84, 89, 96 years). The remaining variables cause of death, educational level, home-ownership, number of children, marital status, single versus multi-person-household, rooms per person and average number of nursing home beds per region were included because of their particular connection with the place of death. To choose the form of the continuous variables of interest, the Bayesian information criterion (BIC) was used, comparing several forms of these variables (linear, square root transformation, categorization). Based on the BIC and for the sake of easy interpretation, we decided to include the number of children in four categories (0, 1–2, 3 or more and an unknown number), the number of rooms in the household per person in three categories (0−< = 1.5, >1.5−< = 2.5 and more than 2.5 rooms) and the number of available beds in a nursing home as a continuous variable. We also tested if interaction terms improved the model which was not the case. Global p-values for each covariate in the final model were obtained using a likelihood ratio test. The level of significance was chosen to be α = 0.05 (two sided tests). Note that no correction for multiple testing was introduced to the model coefficients. All analyses were performed using STATA version 12.1 (StataCorp LP, College Station, Texas, 2011).

## Results

### Descriptive analysis

Tables 1 and 2 present the gender-specific characteristics of our study population.

**Table 1:** Study population characteristics, men.

			hospital	hospital	nursing
	Total N	at home	short	long	home
Deaths	(39,798)	(11,008)	(4,634)	(13,723)	(10,433)
%		*27.7*	*11.6*	*34.5*	*26.2*
Age (mean)		80.3	80.0	79.7	85.5
Nationality*: Swiss nationals (%)	36,874	*28.0*	*11.5*	*33.5*	*27.0*
Nationality*: Non-Swiss (%)	2,924	*24.1*	*13.4*	*46.4*	*16.1*
Cause of death: cancer (%)	11,886	*26.1*	*10.0*	*47.9*	*16.0*
Cause of death: coronary heart disease (%)	6,954	*41.1*	*13.2*	*22.1*	*23.6*
Cause of death: COPD (%)	1,678	*28.1*	*11.7*	*30.2*	*30.0*
Cause of death: dementia (%)	2,136	*15.5*	*0.8*	*9.8*	*73.8*
Cause of death: stroke (%)	2,514	*14.8*	*13.8*	*34.7*	*36.6*
Cause of death: other (%)	14,630	*26.5*	*13.4*	*33.5*	*26.5*
Educational level*: no (%)	1,221	*26.1*	*12.1*	*34.5*	*27.3*
Educational level*: low (%)	10,264	*27.7*	*11.3*	*33.7*	*27.2*
Educational level*: medium (%)	15,744	*28.0*	*12.2*	*35.1*	*24.7*
Educational level*: high (%)	6,814	*30.1*	*11.4*	*36.0*	*22.5*
Educational level*: unknown (%)	5,755	*23.9*	*11.0*	*32.2*	*32.9*
Home-ownership*: Owner-occupiers (%)	18,472	*31.2*	*11.7*	*33.3*	*23.8*
Home-ownership*: Tenants (%)	21,326	*24.6*	*11.6*	*35.5*	*28.3*
Number of children*: 0 (%)	5,861	*25.9*	*11.2*	*34.1*	*28.8*
Number of children*: 1–2 (%)	16,499	*26.7*	*12.1*	*36.8*	*24.4*
Number of children*: >3 (%)	15,018	*29.8*	*11.5*	*32.6*	*26.1*
Number of children*: unknown (%)	2,420	*25.0*	*10.5*	*30.9*	*33.6*
Marital status: never married (%)	2,735	*28.6*	*10.7*	*28.5*	*32.2*
Marital status: married (%)	25,694	*29.5*	*12.6*	*37.7*	*20.2*
Marital status: widowed (%)	9,082	*22.4*	*9.5*	*26.7*	*41.4*
Marital status: divorced (%)	2,287	*26.3*	*11.2*	*36.6*	*25.9*
Single-person-household* (%)	8,205	*24.4*	*9.7*	*29.3*	*36.5*
Multi-person-household* (%)	31,593	*28.5*	*12.1*	*35.8*	*23.5*
Rooms per person*: 0−< = 1.5 (%)	14,653	*26.3*	*12.0*	*35.7*	*26.0*
Rooms per person*: >1.5−< = 2.5 (%)	16,247	*28.9*	*11.9*	*35.3*	*23.9*
Rooms per person*: >2.5 (%)	8,898	*27.6*	*10.5*	*30.9*	*31.0*
Language region*: german (%)	28,747	*29.7*	*11.4*	*31.2*	*27.7*
Language region*: french (%)	9,270	*22.3*	*12.2*	*43.4*	*22.2*
Language region*: italian (%)	1,781	*23.1*	*12.5*	*41.2*	*23.2*
Average number of nursing home beds					
per 100 inhabitants above65 years per (per 106 regions)		6.8	6.7	6.6	7.1

Percentages in italic.

Data source: Swiss Federal Statistical Office, MedStat/SOMED/SNC.

*2000 census data.

**Table 2:** Study population characteristics, women.

			hospital	hospital	nursing
	Total N	at home	short	long	home
Deaths	(45,331)	(9,267)	(3,900)	(11,992)	(20,172)
%		*20.4*	*8.6*	*26.5*	*44.5*
Age (mean)		83.9	82.7	81.6	88.0
Nationality*: Swiss nationals (%)	43,317	*20.4*	*8.6*	*25.9*	*45.1*
Nationality*: Non-Swiss (%)	2,014	*20.8*	*10.0*	*37.9*	*31.3*
Cause of death: cancer (%)	9,351	*20.1*	*6.9*	*47.6*	*25.4*
Cause of death: coronary heart disease (%)	6,865	*27.8*	*9.5*	*16.6*	*46.1*
Cause of death: COPD (%)	1,132	*22.7*	*11.4*	*25.4*	*40.5*
Cause of death: dementia (%)	4,309	*13.4*	*0.6*	*4.7*	*81.3*
Cause of death: stroke (%)	3,979	*13.0*	*11.1*	*26.9*	*49.0*
Cause of death: other (%)	19,695	*21.0*	*10.2*	*24.6*	*44.3*
Educational level*: no (%)	2,159	*20.0*	*7.8*	*25.5*	*46.7*
Educational level*: low (%)	20,569	*19.8*	*8.6*	*25.9*	*45.6*
Educational level*: medium (%)	12,539	*21.7*	*8.8*	*28.0*	*41.5*
Educational level*: high (%)	1,676	*23.4*	*8.7*	*33.0*	*35.0*
Educational level*: unknown (%)	8,388	*19.7*	*8.5*	*24.4*	*47.5*
Home-ownership*: Owner-occupiers (%)	16,839	*24.1*	*9.0*	*27.0*	*39.9*
Home-ownership*: Tenants (%)	28,492	*18.3*	*8.4*	*26.1*	*47.2*
Number of children*: 0 (%)	7,815	*19.3*	*7.9*	*25.8*	*47.0*
Number of children*: 1–2 (%)	18,144	*19.5*	*8.8*	*28.4*	*43.3*
Number of children*: >3 (%)	15,836	*22.2*	*8.7*	*25.6*	*43.5*
Number of children*: unknown (%)	3,536	*19.5*	*8.7*	*21.9*	*49.9*
Marital status: never married (%)	4,489	*20.3*	*7.7*	*23.8*	*48.3*
Marital status: married (%)	9,469	*23.9*	*11.7*	*38.4*	*26.1*
Marital status: widowed (%)	28,109	*19.3*	*7.7*	*22.4*	*50.6*
Marital status: divorced (%)	3,264	*20.5*	*8.8*	*30.6*	*40.2*
Single-person-household* (%)	25,855	*18.7*	*7.5*	*22.8*	*50.9*
Multi-person-household* (%)	19,476	*22.7*	*10.0*	*31.3*	*36.0*
Rooms per person*: 0−< = 1.5 (%)	10,737	*20.4*	*9.0*	*29.1*	*41.5*
Rooms per person*: >1.5−< = 2.5 (%)	14,787	*20.6*	*9.4*	*27.6*	*42.4*
Rooms per person*: >2.5 (%)	19,807	*20.3*	*7.8*	*24.2*	*47.7*
Language region*: german (%)	32,699	*21.7*	*8.5*	*24.7*	*45.1*
Language region*: french (%)	10,545	*17.5*	*8.7*	*31.2*	*42.6*
Language region*: italian (%)	2,087	*15.8*	*9.6*	*30.0*	*44.7*
Average number of nursing home beds					
per 100 inhabitants above65 years (per 106 regions)		6.9	6.8	6.6	7.0

Percentages in italic.

Data source: Swiss Federal Statistical Office, MedStat/SOMED/SNC.

*2000 census data.

Table 1 and table 2 depict the characteristics of the study population stratified by the location of death for men and women, separately. 72% of all men and 80% of all women died in an institution. Almost half of all women (44.5%) and slightly more than one out of four men (26.2%) died in a nursing home. Persons with dementia had the highest percentage of death in nursing homes (74% for men and 81% for women). A higher educational level was associated with a higher proportion of deaths at private homes. Homeowners died more often at home than tenants and so did married men and women compared to never married, divorced and widowed individuals. Furthermore, individuals living in a single-person household were more likely to die in an institution compared to those living in a multi-person household.

### Multivariate analysis

**Figure 2:**
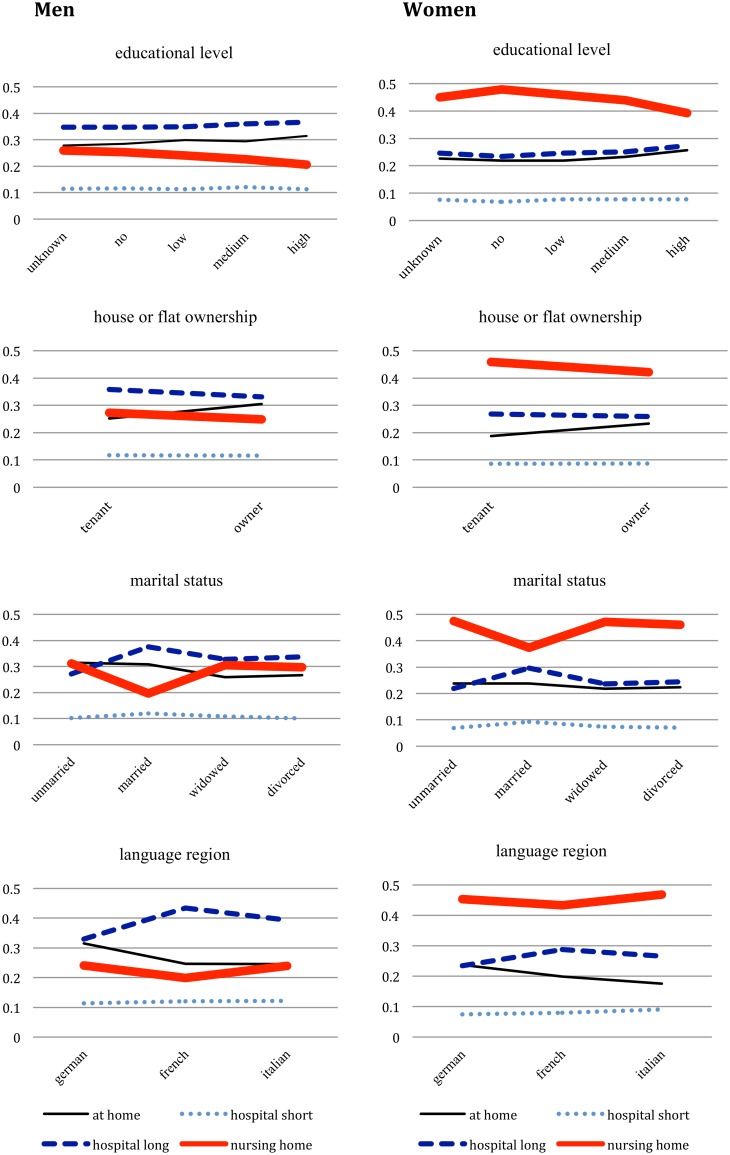
Predicted probabilities to die in one of four types of places of death for selected independent variables. Legend: The probabilities add up to 1 per category of the independent variable which means they can be interpreted as percent. One example: the probability to die in a nursing home compared to other places of death for women is higher for all educational levels compared to other places of death, but it is highest for those with no formal education. Data source: Swiss Federal Statistical Office, MedStat/SOMED/SNC.

The estimated relative risk ratios (RRR) of men and women to die at the three different places of death are presented in Table 3 with death at home as reference category, along with 95% confidence intervals and a joint p-value for each variable from a likelihood ratio test. Note that the p-values were of the same magnitude in men and women, for which reason we only provide one p-value for each variable. For better understanding of the estimated relative risk ratios, Figure 2 presents a series of predicted probability plots for selected variables for both, men and women. Other variables in the model are kept constant at their mean value or their reference category, respectively.

**Table 3:** Results of the multinomial logistic regression analysis for four different places of death (reference value: death at home), Switzerland, 2007 & 2008, individuals born before 1942.

	hospital short				hospital long				nursing home			
	men		women		men		women		men		women	
	RRR *conf. Int*		RRR *conf. Int*		RRR *conf. Int*		RRR *conf. Int*		RRR *conf. Int*		RRR *conf. Int*	
**Cause of death** (p<0.001)												
*Cancer condition (reference)*	*1.00*		*1.00*		*1.00*		*1.00*		*1.00*		*1.00*	
coronary heart disease	0.87	*0.78–0.96*	1.13	*0.99–1.29*	0.30	*0.28–0.33*	0.29	0.26–0.32	0.64	0.59–0.70	0.81	0.74–0.88
COPD	1.10	*0.92–1.31*	1.48	*1.17–1.86*	0.59	*0.51–0.67*	0.47	0.39–0.56	1.27	1.10–1.47	1.09	0.92–1.29
dementia	0.13	*0.08–0.22*	0.14	*0.09–0.21*	0.33	*0.28–0.40*	0.16	0.13–0.19	5.26	4.59–6.03	3.04	2.72–3.40
stroke	2.49	*2.12–2.93*	2.73	*2.33–3.20*	1.30	*1.14–1.48*	0.97	0.86–1.09	2.72	2.37–3.12	1.91	1.70–2.16
other	1.35	*1.23–1.47*	1.59	*1.42–1.77*	0.69	*0.65–0.74*	0.55	0.51–0.59	1.09	1.01–1.18	1.05	0.98–1.14
**Educational level** (p<0.001)												
*medium (ref.)*	*1.00*		*1.00*		*1.00*		*1.00*		*1.00*		*1.00*	
unknown	1.02	*0.90–1.14*	1.00	*0.88–1.12*	1.02	*0.94–1.11*	1.01	0.92–1.10	1.22	1.11–1.34	1.05	0.97–1.14
no	1.00	*0.81–1.22*	0.92	*0.76–1.12*	0.99	*0.85–1.16*	0.99	0.86–1.14	1.16	0.97–1.37	1.16	1.02–1.32
low	0.92	*0.84–1.01*	1.05	*0.95–1.15*	0.95	*0.89–1.02*	1.04	0.98–1.12	1.05	0.98–1.13	1.11	1.04–1.18
high	0.88	*0.80–0.98*	0.89	*0.73–1.10*	0.95	*0.88–1.02*	0.99	0.86–1.14	0.85	0.78–0.93	0.81	0.70–0.93
**House or flat owner** (p<0.001)												
*tenant (ref.)*	*1.00*		*1.00*		*1.00*		*1.00*		*1.00*		*1.00*	
owner-occupier	0.82	*0.76–0.88*	0.80	*0.74–0.88*	0.76	*0.72–0.80*	0.77	0.73–0.82	0.73	0.69–0.78	0.72	0.68–0.77
**Children** (p<0.001)												
*0 (ref.)*	1.00		1.00		1.00		1.00		1.00		1.00	
1–2	0.98	*0.87–1.11*	1.05	*0.92–1.19*	0.91	*0.83–0.99*	1.00	0.92–1.10	0.89	0.81–0.99	0.96	0.88–1.04
>3	0.88	*0.78–1.00*	0.92	*0.81–1.05*	0.79	*0.72–0.87*	0.85	0.77–0.94	0.84	0.76–0.93	0.86	0.79–0.94
unknown	0.94	*0.78–1.12*	1.08	*0.91–1.29*	0.90	*0.78–1.03*	0.86	0.75–0.98	0.96	0.83–1.10	0.94	0.84–1.06
**Marital status** (p<0.001)												
*married (ref.)*	*1.00*		*1.00*		*1.00*		*1.00*		*1.00*		*1.00*	
never married	0.84	*0.70–1.01*	0.74	*0.62–0.89*	0.71	*0.62–0.81*	0.74	0.65–0.85	1.55	1.34–1.79	1.28	1.13–1.44
widowed	1.07	*0.96–1.19*	0.86	*0.77–0.96*	1.04	*0.96–1.13*	0.87	0.80–0.95	1.84	1.69–1.99	1.38	1.27–1.50
divorced	0.97	*0.81–1.15*	0.81	*0.68–0.96*	1.04	*0.91–1.18*	0.87	0.77–0.99	1.74	1.51–2.01	1.31	1.15–1.48
**Living condition** (p<0.001)												
*Single-person household (ref.)*	*1.00*		*1.00*		*1.00*		*1.00*		*1.00*		*1.00*	
Multi-person household	1.11	*0.97–1.27*	0.97	*0.86–1.10*	1.04	*0.94–1.15*	0.94	0.86–1.03	0.87	0.79–0.97	0.81	0.75–0.88
**Rooms per person** (p<0.05)												
*0*−*< = 1.5 (ref.)*	1.00		1.00		1.00		1.00		1.00		1.00	
>1.5−< = 2.5	0.98	*0.91–1.07*	1.11	*1.00–1.23*	1.02	*0.96–1.08*	1.00	0.92–1.08	0.93	0.87–1.00	0.97	0.90–1.05
>2.5	0.98	*0.87–1.11*	0.99	*0.87–1.13*	1.00	*0.91–1.09*	0.97	0.88–1.07	0.86	0.78–0.95	0.86	0.79–0.94
**Language region** (p<0.001)												
*german (ref.)*	*1.00*		*1.00*		*1.00*		*1.00*		*1.00*		*1.00*	
french	1.37	*1.25–1.50*	1.28	*1.16–1.42*	1.69	*1.58–1.81*	1.47	1.36–1.58	1.06	0.98–1.15	1.14	1.06–1.22
italian	1.38	*1.16–1.64*	1.65	*1.37–1.99*	1.53	*1.35–1.75*	1.54	1.33–1.77	1.28	1.10–1.49	1.40	1.22–1.60
**NH beds density** * (p<0.001)	0.99	*0.97–1.01*	1.00	*0.98–1.03*	0.98	*0.97–0.99*	0.97	0.96–0.99	1.07	1.05–1.09	1.04	1.03–1.06

RRR = relative risk ratios, conf. interval = 95% conf. interval, p-values from likelihood ratio tests.

model also included control variablHes nationality and age as cubic spline, results not shown.

* = average number of nursing home beds per 100 habitants above 65 years (per 106 regions).

Data source: Swiss Federal Statistical Office, MedStat/SOMED/SNC.

Older persons of both sexes were more likely to die in nursing homes than comparatively younger people within the age group over 65 years. We found significant differences between causes of death. In comparison to patients with malignant neoplasms (reference category), persons with dementia had a significantly higher risk of dying in a nursing home than dying at home (men: RRR 5.26, CI 4.59–6.03; women RRR 3.04, CI 2.72–3.40). In contrast, persons with coronary heart disease had a significantly lower relative risk for dying in any institution than for dying at home. The estimated coefficients and graphs show that a higher educational level slightly reduced the relative risk to die in nursing homes compared to a medium educational level (men: RRR 0.85, CI 0.78–0.93; women: RRR 0.81, CI 0.70–0.93). Homeowners died more often at home than in an institution.

Having many children prevents dying in an institution: Having at least three children compared to no children, for instance, decreased the risk ratio for dying in a nursing home compared to death at home significantly (men: RRR 0.84, CI 0.76–0.93; women: RRR 0.86, CI 0.79–0.94). Being never married increased the relative risk of dying in a nursing home in compared to dying at home (men: RRR 1.55, CI 1.34–1.79; women: RRR 1.28, CI 1.13–1.44), and decreases the relative risk of dying in a hospital after a long stay (men: RRR 0.71, CI 0.62–0.81; women: RRR 0.74, CI 0.65–0.85). Being widowed or divorced had different effects for men and women: Compared to being married, it significantly increases the relative risk ratios of dying in a nursing for both men and women, but the respective relative risk ratio for death in a hospital was only significant for women. Living in a multi-person household compared to living alone decreased the relative risks for dying in a nursing home (men: RRR 0.87, CI 0.79–0.97; women: RRR 0.81, CI 0.75–0.88). The higher the number of rooms in a household per person, the higher the relative risk of dying at home compared to dying in a nursing home. We found for instance a significance effect for 2.5 rooms or more per person compared to 0–1.5 rooms per person (men: RRR 0.86, CI 0.78–0.95; women: RRR 0.86, CI 0.79–0.94).

In both the French and the Italian speaking part of Switzerland, the relative risks of dying in an institution rather than at home were significantly higher than in the German speaking part. We found, for instance, higher relative risks of hospital deaths after a longer stay in the French speaking part (men RRR 1.69, CI 1.58–1.81; women: RRR 1.47, CI 1.36–1.58) and Italian speaking part (men RRR 1.53, CI 1.35–1.75; women RRR 1.54, CI 1.33–1.77) compared to the German speaking part, which is also illustrated in Figure 2. The availability of nursing home beds showed the expected effect: the higher the density of beds, the higher the relative risk of dying in a nursing home (men: RRR 1.07, CI 1.05–1.09; women: RRR 1.04, CI 1.03–1.06).

## Discussion

We found substantial socio-demographic variation concerning the place of death. The place of death was not determined by underlying somatic diseases alone, but also by a variety of socio- demographic and familial/housing determinants, with a different impact on men and women. While hospital deaths appeared to be mostly due to medical reasons, dying in a nursing home was mainly determined by socio-demographic, familial and regional characteristics. Persons with a low SEP had higher relative risks of dying in a nursing home compared to dying at home. This is in line with a study from Belgium. [2] Considering homeownership and the number of rooms per person as other proxies for SEP confirmed this pattern. One explanation for this may be that people with a higher SEP have generally a better health status and need therefore less nursing care than people with a lower SEP. Another explanation could be that people with higher SEP can afford home nursing care more frequently. More empirical research is needed to explore this issue.

In our study population, nearly 45% of women died in a nursing home compared to 26% of men. In spite of a general wish to die at home, [4] only around 28% of men and 20% of women died at home. These results are in line with precedent studies from Switzerland following another approach [16] and comparable with studies from other countries. [2], [21]–[23], [33], [34] The gender difference is most likely due to the longer life expectancy of women. Men live more often in a multi-person household, possibly together with a younger female partner. In contrast, women more often survive their spouses or stay divorced. We therefore hypothesize that men often continue to live in their private home, even when their health status is getting worse and health care is needed, whereas single women with similar health status are placed in a nursing home.

Among those dying in a hospital, there were noticeable differences between persons with longer and short (≤2 days) stays preceding death. Relative to cancer deaths, individuals deceased due to stroke or respiratory diseases were more likely to have died after a short hospitalisation than at home, whereas the corresponding effect was not significant or reversed for long-term hospitalisations for stroke and COPD, respectively. We suppose that short hospital stays preceding deaths are often due to acute exacerbation of a pre-existing disease. Familial factors were also important: Having children, being married and living with a spouse or any other person increased the relative risks of dying at home, which is probably more in accordance with the patient’s and his relatives’ wishes, [3], [7] unless it becomes too stressful for the caregivers. [9] On the other hand, being never married, widowed or divorced and living alone increases the relative risks to die in a nursing home. Dying in nursing homes could become even more frequent in the future due to the aging of the population: the older the people, the higher the probability to die in a nursing home instead of a hospital. [34], [35] As a consequence, SEP and familial/housing factors could gain even more influence on the place of end-of-life.

The analysis of the three Swiss language regions revealed that death at home was significantly more likely for both men and women in the German speaking than in the French or Italian speaking part. The reason might be cultural differences regarding attitudes towards death and life-prolonging measures, as described for physicians from the three Swiss language areas. [36] We therefore assume that people in the German speaking part more strongly prefer to die at home, especially when the underlying disease is incurable. In a study on main reasons for institutionalization in Switzerland, [37] inability of housekeeping was the most frequent answer, even before medical reasons or need for support.

### Strengths and limitations

The strength of our study is the national comprehensiveness and the size of the study population, which minimizes selections bias. We were able to explore a wide variety of socio- demographic characteristics based on information individually linked from the census.

The study has two main limitations: First, we assumed that all deaths not tracked in hospitals or nursing homes occurred at home. Second, we were not able to link all deaths to a census record. This problem was more prevalent in nursing homes (ca. 12%) than in hospitals (ca. 5%). However, linkage success did not substantially vary by region of living, age or sex of the deceased persons. A further limitation was the lack of actual information about income, assets and health insurance coverage of the study population. Our results emphasize the importance of nursing homes as places of death in Switzerland. Our findings might therefore be generalizable foremost to countries with a similar socio-economic setting (e. g., The Netherlands, Norway, Iceland) [34].

### Conclusion

Death at home is an unaccomplished wish for a majority of elderly people in Switzerland. Dying in an institution is rather the norm than exception – and this makes it important to scrutinize the reasons why people die where they do. Dying in a hospital appears to be mainly driven by medical reasons. In contrast, living and dying in a nursing home is substantially driven by socio-demographic determinants such as SEP, living alone, housing characteristics and structural (regional) factors. The proportion of deaths occurring in nursing homes may increase in the future [34], [35] and as a consequence also the importance of sociodemographic determinants of living conditions at the end-of-life. Individuals with a lower SEP, living alone or having no children, i.e., those with less resources and therefore less freedom of choice are overrepresented in nursing homes. Thus, health policy should aim to empower these groups when having to decide whether to move to a nursing home or to stay in a private home. Since nursing home stays are rather expensive, more public engagement in home care offers may not only meet the request of many concerned persons but also pay off in a macroeconomic view. The interplay between demand and need, between availability of nursing home and hospital beds as well as home care offers, is complex and so are the various push and pull effects exerted by the stakeholders [38].
